# Supplementary material for: Nanoparticle display of prefusion coronavirus spike elicits S1-focused cross-reactive antibody response against diverse coronavirus subgenera
Source: Nat Commun. 2023 Oct 4;14:6195. doi: 10.1038/s41467-023-41661-4 (PMC10551005; doi:10.1038/s41467-023-41661-4)
Supplement: Supplementary file 1 — Supplementary Information [file 41467_2023_41661_MOESM1_ESM.pdf]

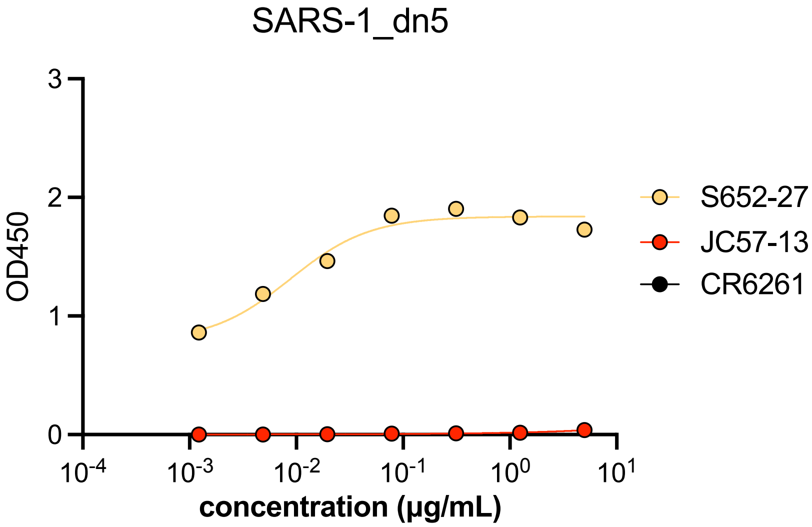

Supplementary Fig. 1: MERS-specific monoclonal antibody JC57-13 does not bind purified SARS-1_dn5.

For further validation, ELISAs were performed on SARS-1_dn5 nanoparticles using monoclonal antibodies specific for SARS (S652-27), MERS (JC57-13), and negative control, influenza-HA (CR6261).

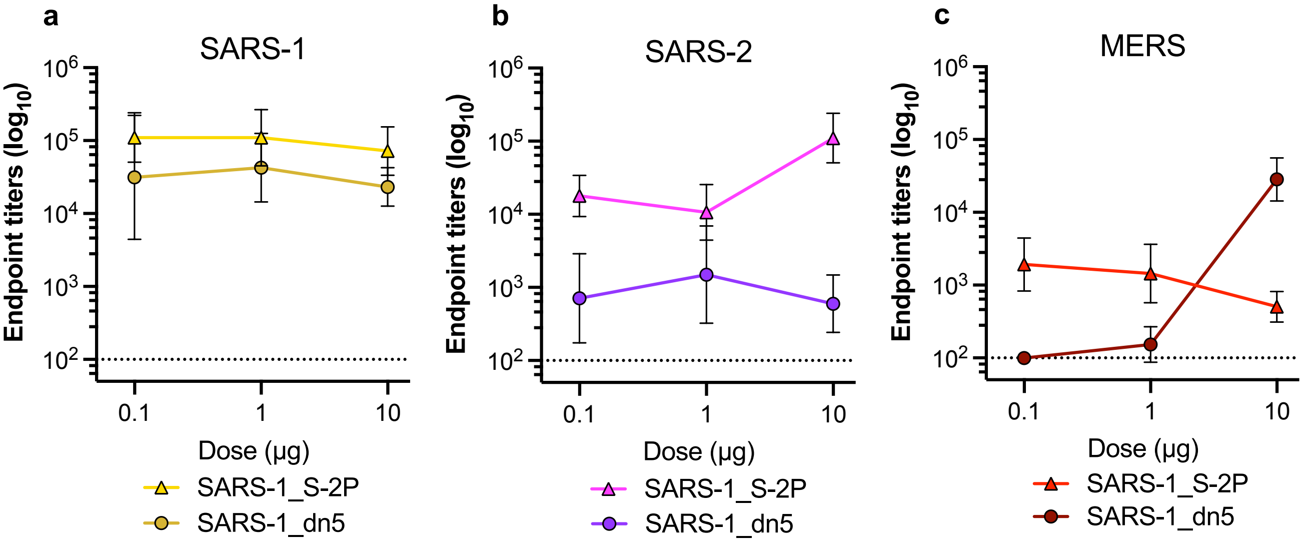

Supplementary Fig. 2: Dose escalation.

a-c Groups of N=10 C57BL/6 mice were immunized with 0.1, 1.0, or 10µg of SARS-1_S-2P or SARS-1_dn5 and bled for serology 3 weeks later. Sera were screened by ELISA for binding to SARS-1_S-2P, SARS-2_S-2P, or MERS_S-2P. symbols represent geometric means with geometric standard deviation.

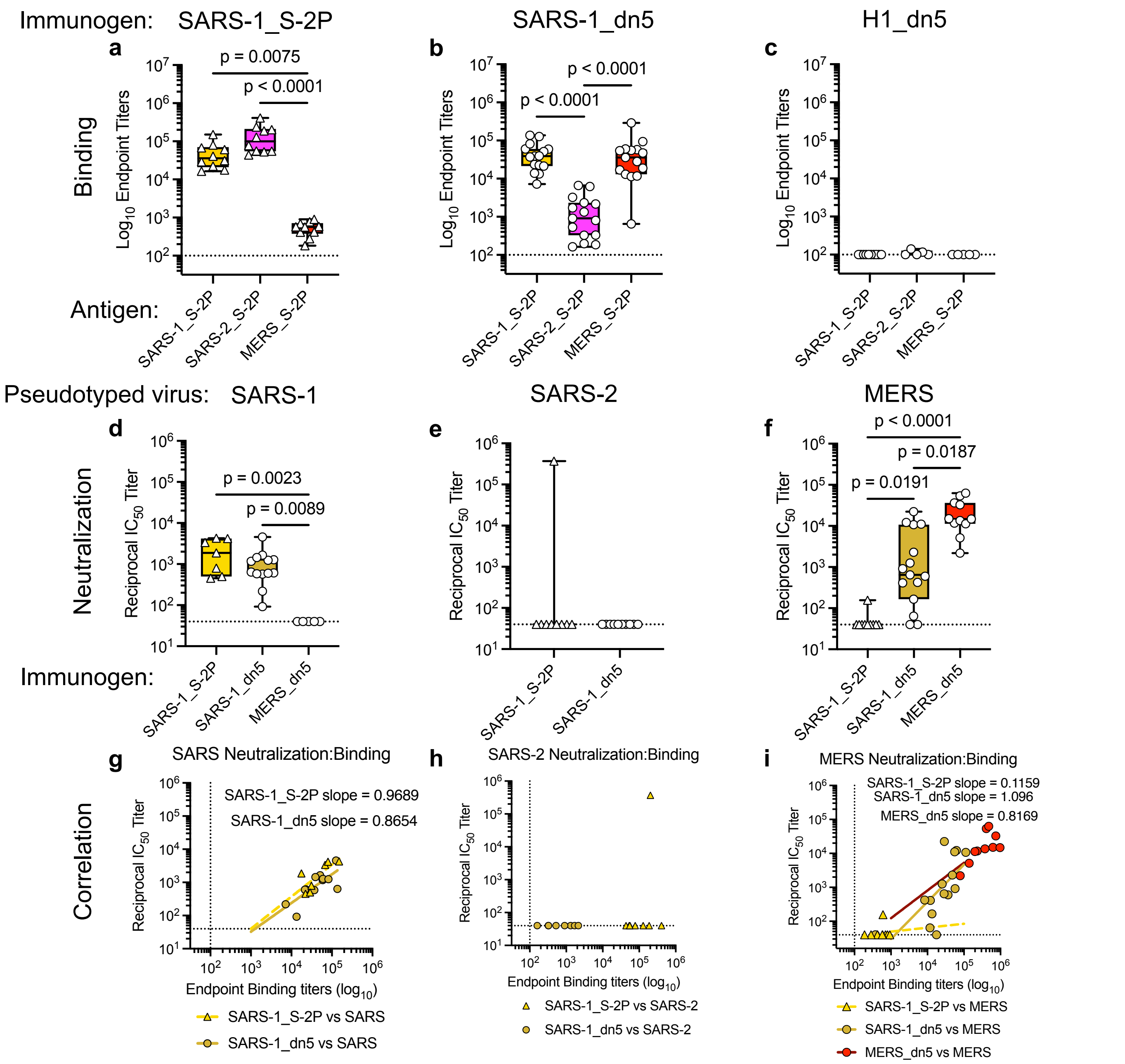

Supplementary Fig. 3: Assembly of SARS-1_S-2P on dn5 elicits potent cross-neutralizing antibodies. **a-f** Groups of 5-10 female C57BL/6J mice from two independent, concatenated studies were immunized at weeks 0 and 3 with 10 µg of SARS-1_S-2P as a soluble trimer (N=10) or displayed on dn5 particles (N=15) with SAS adjuvant and bled at week 5 for serology. Control mice were immunized with H1_dn5 (N=10). **a-c** Serum was screened for binding by ELISA to SARS-1_, SARS-2_, and MERS_S-2P. **d-f** Serum was then assessed for its capacity to neutralize SARS-1, SARS-2, and MERS pseudotyped viruses. **g-i** To plot the potency of neutralizing antibodies, correlation plots of binding (x-axis) to neutralization (y-axis) where the slope (neutralization/binding) indicates the ratio of neutralizing to binding antibody titers were generated. Boxes and horizontal bars denote the interquartile range (IQR) and medians, respectively. Whisker endpoints are equal to the minimum and maximum values. Statistical analysis was performed using non-parametric Kruskal-Wallis test with Dunn's multiple comparisons. *P<0.05, ***P* < 0.01, *****P* < 0.0001.

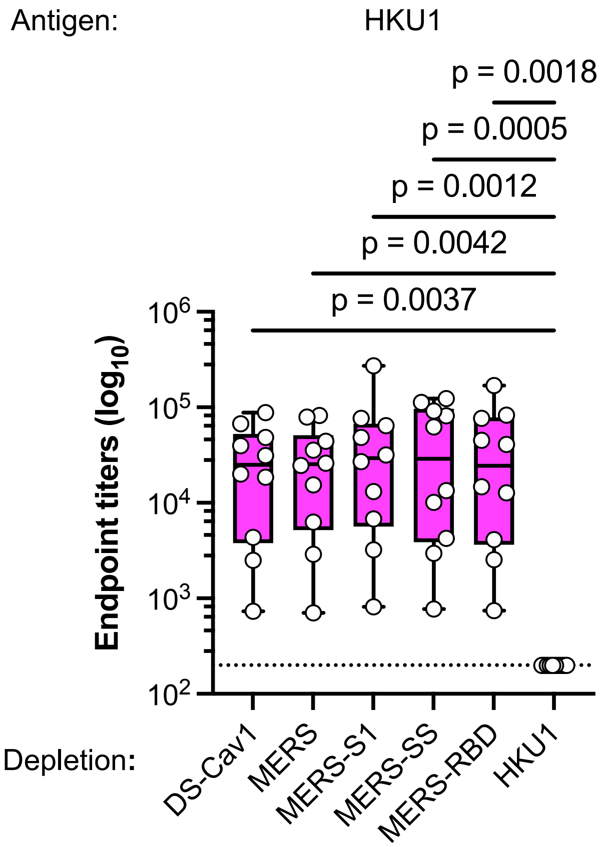

Supplementary Fig. 4: SARS-1_dn5 sera depleted with MERS-CoV spike domains binds to HKU1 spike.

To elucidate cross-reactive domain specificity, sera from SARS-1_dn5-immunized mice (N=10) were depleted with MERS_S-2P and its domains, S1, SS, and RBD then screened for residual binding to HKU1. Boxes and horizontal bars denote the IQR and medians, respectively. Whisker endpoints are equal to the minimum and maximum values. Circles denote each individual animal. Statistical analysis was performed using non-parametric Kruskal-Wallis test with Dunn's multiple comparisons. $^{**}P < 0.01$, $^{***}P < 0.001$, $^{****}P < 0.0001$

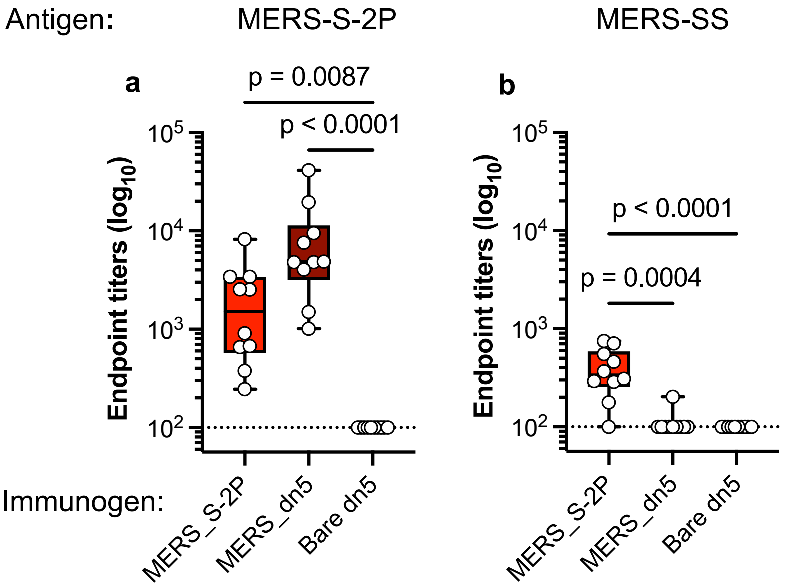

Supplementary Fig. 5: Nanoparticle assembly restricts access to S2 and targets S1 domain of spike.

a-b N=10 mice were immunized once with 10 µg of MERS_S-2P or MERS_dn5 and bled at week 3. Sera was tested for antibody binding to MERS_S-2P and MERS_SS. Boxes and horizontal bars denote the IQR and medians, respectively. Whisker endpoints are equal to the minimum and maximum values. Circles denote each individual animal. Statistical analysis was performed using non-parametric Kruskal-Wallis test with Dunn's multiple comparisons. $^{**}P < 0.01$, $^{***}P < 0.001$, $^{****}P < 0.0001$.

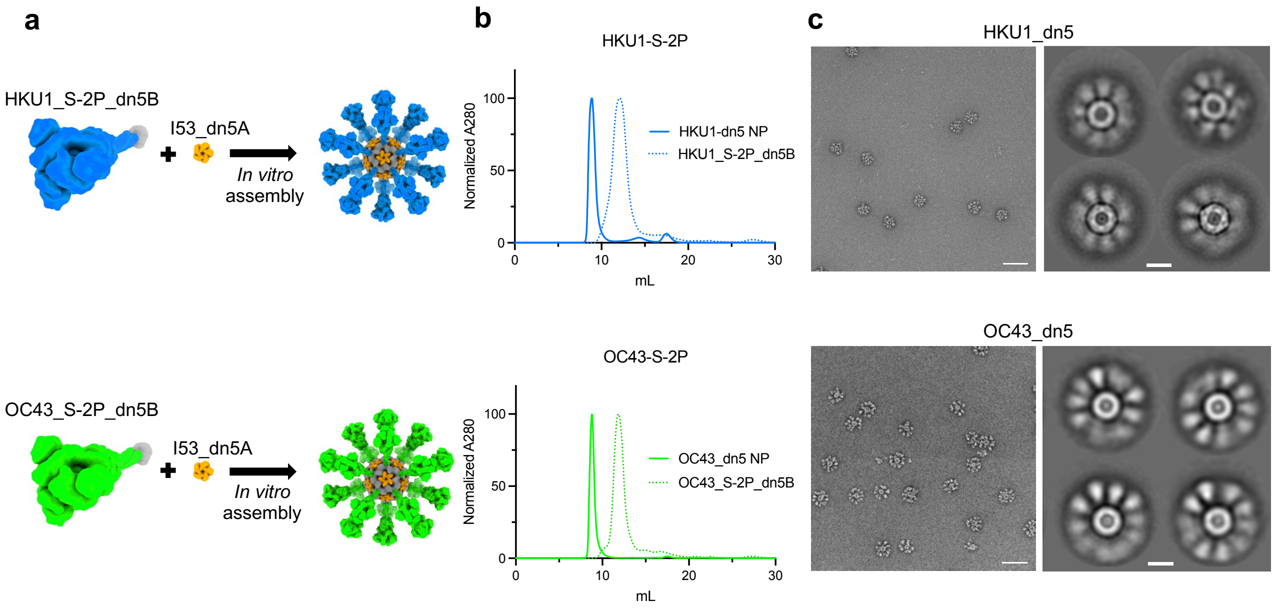

Supplementary Fig. 6: Design and characterization of CoV-S2-P displayed on I53-dn5.
a Computer-generated models of prefusion-stabilized spike trimers (S-2P) from HKU1 and OC43 and their homotypic display on I53-dn5 nanoparticle. Icosahedral nanocage displays 20 trimers. **b** Trace profiles of S-2P-dn5b trimer and -dn5 nanocage purification by size exclusion chromatography. **c** Representative images of CoV-S-2P_dn5 at 50,000-57,000x magnification and 2D class averages. Scale bars correspond to 100 nm (representative images) and 20 nm (2D class averages).

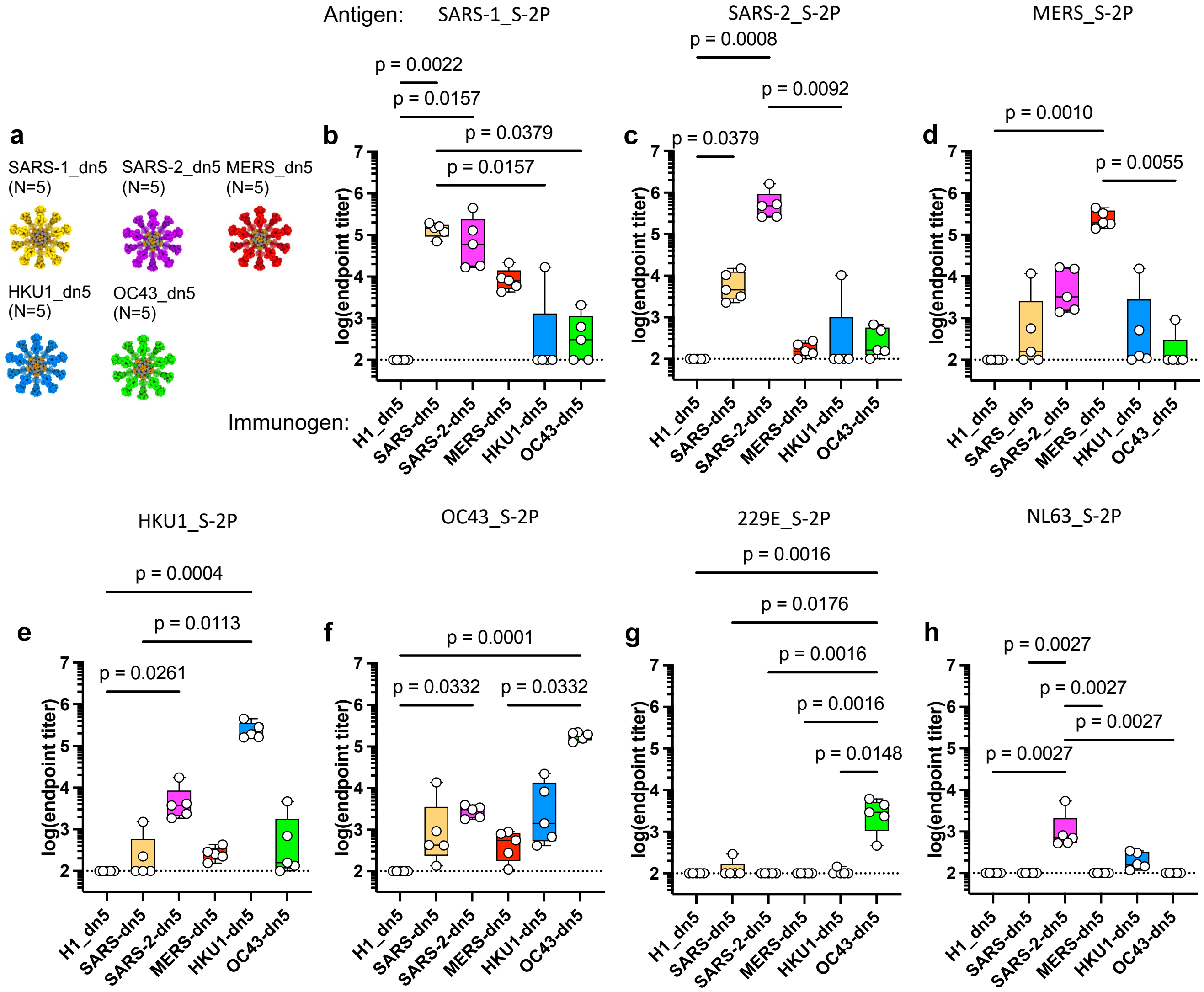

Supplementary Fig. 7: dn5 elicits broadly cross-reactive antibodies from diverse CoV-spikes.
a Groups of N=5 C57BL/6 female mice were immunized twice at weeks 0 and 3 with 10 µg of SARS-1_dn5, SARS-2_dn5, MERS_dn5, HKU1_dn5, or OC43_dn5 nanoparticles and bled at week 5. Control mice were immunized with H1_dn5. **b-h** Sera was screened by ELISA for antibody binding to SARS_S-2P, SARS-2_S-2P, MERS_S-2P, HKU1_S-2P, OC43_S-2P, 229E_S-2P, and NL63_S-2P. Boxes and horizontal bars denote the IQR and medians, respectively. Whisker endpoints are equal to the minimum and maximum values. Circles denote each individual animal.

Sequence List:
MERS_S-2P:
MIHSVFLLMFLLTPTESYVDVGPDSVKSACIEVDIQQTFFDKTWPRPIDVSKADGIIYPQGRTYSNITITYQGLFP
YQGDHGDMYVYSAGHATGTTPQKLFVANYSQDVKQFANGFVVRIGAAANSTGTVIISPSTSATIRKIYPAFML
GSSVGNFSDGKMGRFFNHTLVLLPDGCGTLLRAFYCILEPRSGNHCPAGNSYTSFATYHTPATDCSDGNYNRN
ASLNSFKEYFNLRNCTFMYTYNITEDEILEWFGITQTAQGVHLFSSRYVDLYGGNMFQFATLPVYDTIKYYSIIPH
SIRSIQSDRKAWAAFYVYKLQPLTFLLDFSVDGYIRRAIDCGFNDLSQLHCSYESFDVESGVYSVSSFEAKPSGSV
VEQAEGVECDFSPLLSGTPPQVYNFKRLVFTNCNYNLTKLLSLFSVNDFTCSQISPAAIASNCYSSLILDYFSYPLS
MKSDLSVSSAGPISQFNYKQSFSNPTCLILATVPHNLTTITKPLKYSYINKCSRFLSDDRTEVPQLVNANQYSPCV
SIVPSTVWEDGDYYRKQLSPLEGGGWLVASGSTVAMTEQLQMGFGITVQYGTDTNSVCPKLEFANDTKIASQ
LGNCVEYSLYGVSGRGVFQNCTAVGVRQQRFVYDAYQNLVGYYSDDGNYYCLRACVSVPVSVIYDKETKTHAT
LFGSVACEHISSTMSQYSRSTRSMLKRRDSTYGPLQTPVGCVLGLVNSSLFVEDCKLPLGQSLCALPDTPSTLTP
ASVGSVPGEMRLASIAFNHPIQVDQLNSSYFKLSIPTNFSFGVTQEYIQTTIQKVTVDCKQYVCNGFQKCEQLL
REYGQFCSKINQALHGANLRQDDSVRNLFASVKSSQSSPIIPGFGGDFNLTLLEPVSISTGSRSARSAIEDLLFDK
VTIADPGYMQGYDDCMQQGPASARDLICAQYVAGYKVLPPLMDVNMEAAYTSSLLGSIAGVGWTAGLSSFA
AIPFAQSIFYRLNGVGITQQVLSENQKLIANKFNQALGAMQTGFTTTNEAFHKVQDAVNNNAQALSKLASEL
SNTFGAISASIGDIIQRLDPPEQDAQIDRLINGRLTTLNAFVAQQLVRSESAALSAQLAKDKVNECVKAQSKRSG
FCGQGTHIVSFVVNAPNGLYFMHVGYYPSNHIEVVSAYGLCDAANPTNCIAPVNGYFIKTNNTRIVDEWSYT
GSSFYAPEPITSLNTKYVAPQVTYQNISTNLPPPLLGNSTGIDFQDELDEFFKNVSTSIPNFGSLTQINTTLLDLTY
EMLSLQQVVKALNESYIDLKELGNYTYGSGYIPEAPRDGQAYVRKDGEWVLLSTFLGRSLEVLFQGPGHHHH
HHHHSAWSHPQFEKGGGSGGGGSGGSAWSHPQFEK
SARS_S-2P:
MFIFLLFLTLTSGSDLDRCTTFDDVQAPNYTQHTSSMRGVYYPDEIFRSDTLYLTQDLFLPFYSNVTGFHTINHTF
GNPVIPFKDGIYFAATEKSNVVRGWVFGSTMNNKSQSVIIINNSTNVVIRACNFELCDNPFFAVSKPMGTQTH
TMIFDNAFNCTFEYISDAFSLDVSEKSGNFKHLREFVFKNKDGFLYVYKGYQPIDVVRDLPSGFNTLKPIFKLPL
GINITNFRAILTAFSPAQDIWGTSAAAYFVGYLKPTTFMLKYDENGTITDAVDCSQNPLAELKCSVKSFEIDKGIY
QTSNFRVVPSGDVVRFPNITNLCPFGEVFNATKFPSVYAWERKKISNCVADYSVLYNSTFFSTFKCYGVSATKLN
DLCFSNVYADSFVVKGDDVRQIAPGQTGVIADYNYKLPDDFMGCVLAWNTRNIDATSTGNYNYKYRYLRHG
KLRPFERDISNVPFSPDGKPCTPPALNCYWPLNDYGFYTTTGIGYQPYRVVVLSFELLNAPATVCGPKLSTDLIK
NQCVNFNFNGLTGTGVLTPSSKRFQPFQQFGRDVSDFTDSVRDPKTSEILDISPCAFGGVSVITPGTNASSEVA
VLYQDVNCTDVSTAIHADQLTPAWRIYSTGNNVFQTQAGCLIGAEHVDTSYECDIPIGAGICASYHTVSLLRSTS
QKSIVAYTMSLGADSSIAYSNNTIAIPTNFSISITTEVMPVSMAKTSVDCNMYICGDSTECANLLLQYGSFCTQL
NRALSGIAAEQDRNTREVFAQVKQMYKTPTLKYFGGFNFSQILPDPLKPTKRSFIEDLLFNKVTLADAGFMKQ
YGECLGDINARDLICAQKFNGLTVLPPLLTDDMIAAYTAALVSGTATAGWTFGAGAALQIPFAMQMAYRFNGI
GVTQNVLYENQKQIANQFNKAISQIQESLTTTSTALGKLQDVVNQNAQALNTLVKQLSSNFGAISSVLNDILSR
LDPPEAEVQIDRLITGRLQSLQTYVTQQLIRAAEIRASANLAATKMSECVLGQSKRVDFCGKGYHLMSFPQAA
PHGVVFLHVTYVPSQERNFTTAPAICHEGKAYFPREGVFVFNGTSWFITQRNFFSPQIITTDNTFVSGNCDVVI
GIINNTVYDPLQPELDSFKEELDKYFKNHTSPDVDLGDISGINASVVNIQKEIDRLNEVAKNLNESLIDLQELGKY
EQGSGYIPEAPRDGQAYVRKDGEWVLLSTFLGRSLEVLFQGPGHHHHHHHHSAWSHPQFEK
SARS-2_S-2P:
MFVFLVLLPLVSSQCVNLTTRTQLPPAYTNSFTRGVYYPDKVFRSSVLHSTQDLFLPFFSNVTWFHAIHVSGTN
GTKRFDNPVLPFNDGVYFASTEKSNIIRGWIFGTTLDSKTQSLLIVNNATNVVIKVCEFQFCNDPFLGVYYHKN
NKSWMESEFRVYSSANNCTFEYVSQPFLMDLEGKQGNFKNLREFVFKNIDGYFKIYSKHTPINLVRDLPQGFS
ALEPLVDLPIGINITRFQTLLALHRSYLTPGDSSSGWTAGAAAYYVGYLQPRTFLLKYNENGTITDAVDCALDPLS

ETKCTLKSFTVEKGIYQTSNFRVQPTESIVRFPNITNLCPFGEVFNATRFASVYAWNRKRISNCVADYSVLYNSAS
FSTFKCYGVSPTKLNDLCFTNVYADSFVIRGDEVRQIAPGQTGKIADYNYKLPDDFTGCVIAWNSNNLDSKVG
GNYNYLYRLFRKSNLKPFERDISTEIYQAGSTPCNGVEGFNCYFPLQSYGFQPTNGVGYQPYRVVVLSFELLHA
PATVCGPKKSTNLVKNKCVNFNFNGLTGTGVLTESNKKFLPFQQFGRDIADTTDAVRDPQTLEILDITPCSFGG
VSVITPGTNTSNQVAVLYQDVNCTEVPVAIHADQLTPTWRVYSTGSNVFQTRAGCLIGAEHVNNSYECDIPIG
AGICASYQTQTNSPRRARSVASQSIIAYTMSLGAENSVAYSNNSIAIPTNFTISVTTEILPVSMTKTSVDCTMYIC
GDSTECSNLLLQYGSFCTQLNRALTGIAVEQDKNTQEVFAQVKQIYKTPPIKDFGGFNFSQILPDPSKPSKRSFIE
DLLFNKVTLADAGFIKQYGDCLGDIAARDLICAQKFNGLTVLPPLLTDEMIAQYTSALLAGTITSGWTFGAGAA
LQIPFAMQMAYRFNGIGVTQNVLYENQKLIANQFNSAIGKIQDSLSSTASALGKLQDVVNQNAQALNTLVKQ
LSSNFGAISSVLNDILSRLDPPEAEVQIDRLITGRLQSLQTYVTQQLIRAAEIRASANLAATKMSECVLGQSKRVD
FCGKGYHLMSFPQSAPHGVVFLHVTYVPAQEKNFTTAPAICHDGKAHFPREGVFVSNGTHWFVTQRNFYEP
QIITTDNTFVSGNCDVVIGIVNNTVYDPLQPELDSFKEELDKYFKNHTSPDVDLGDISGINASVVNIQKEIDRLN
EVAKNLNESLIDLQELGKYEQGSGYIPEAPRDGQAYVRKDGEWVLLSTFLGRSLEVLFQGPGHHHHHHHHSA
WSHPQFEK

HKU1_S-2P:

MFLIIFILPTTLAVIGDFNCTNSFINDYNKTIPRISEDVVDVSLGLGTYYVLNRVYLNTTLLFTGYFPKSGANFRDL
ALKGSIYLSTLWYKPPFLSDFNNGIFSKVKNTKLYVNNTLYSEFSTIVIGSVFVNTSYTIVVQPHNGILEITACQYT
MCEYPHTVCKSKGSIRNESWHIDSSEPLCLFKKNFTYNVSADWLYFHFYQERGVFYAYYADVGMPTTFLFSLYL
GTILSHYYVMPLTCNAISSNTDNETLEYWVTPLSRRQYLLNFDEHGVITNAVDCSSSFLSEIQCKTQSFAPNTGV
YDLSGFTVKPVATVYRRIPNLPDCDIDNWLNNVSVPSPLNWERRIFSNCNFNLSTLLRLVHVDSFSCNNLDKSK
IFGSCFNSITVDKFAIPNRRRDDLQLGSSGFLQSSNYKIDISSSSCQLYYSLPLVNVTINNFNPSSWNRRYGFGSF
NLSSYDVVYSDHCFSVNSDFCPCADPSVVNSCAKSKPPSAICPAGTKYRHCDLDTTLYVKNWCRCSCLPDPIST
YSPNTCPQKKVVVGIGEHCPGLGINEEKCGTQLNHSSCFCSPDAFLGWSFDSCISNNRCNIFSNFIFNGINSGT
TCSNDLLYSNTEISTGVCVNYDLYGITGQGIFKEVSAAYYNNWQNLLYDSNGNIIGFKDFLTNKTYTILPCYSGRV
SAAFYQNSSSPALLYRNLKCSYVLNNISFISQPFYFDSYLGCVLNAVNLTSYSVSSCDLRMGSGFCIDYALPSSGG
SGSGISSPYRFVTFEPFNVSFVNDSVETVGGLFEIQIPTNFTIAGHEEFIQTSSPKVTIDCSAFVCSNYAACHDLLS
EYGTFCDNINSILNEVNDLLDITQLQVANALMQGVTLSSNLNTNLHSDVDNIDFKSLLGCLGSQCGSSSRSLLE
DLLFNKVKLSDVGFVEAYNNCTGGSEIRDLLCVQSFNGIKVLPPILSETQISGYTTAATVAAMFPPWSAAAGVP
FSLNVQYRINGLGVTMDVLNKNQKLIANAFNKALLSIQNGFTATNSALAKIQSVVNANAQALNSLLQQLFNKF
GAISSSLQEILSRLDPPEAQVQIDRLINGRLTALNAYVSQQLSDITLIKAGASRAIEKVNECVKSQSPRINFCGNG
NHILSLVQNAPYGLLFIHFSYKPTSFKTVLVSPGLCLSGDRGIAPKQGYFIKQNDSWMFTGSSYYYPEPISDKNV
VFMNSCSVNFTKAPFIYLNNSIPNLSDFEAELSLWFKNHTSIAPNLTFNSHINATFLDLYYEMNVIQESIKSLNSG
RLEVLFQGPGGYIPEAPRDGQAYVRKDGEWVLLSTFLGHHHHHHHHSAWSHPQFEK

229E_S-2P:

MFVLLVAYALLHIAGCQTTNGTNTSHSVCNGCVGHSENVFAVESGGYIPSNFAFNNWFLLTNTSSVVDGVVRS
FQPLLLNCLWSVSGSQFTTGFVYFNGTGRGACKGFYSNASSDVIRYNINFEENLRRGTILFKTSYGAVVFYCTN
NTLVSGDAHIPSGTVLGNFYCFVNTTIGNETTSAFVGALPKTVREFVISRTGHFYINGYRYFSLGDVEAVNFNVT
NAATTVCTVALASYADVLVNVSQTAIANIIYCNSVINRLRCDQLSFDVPDGFYSTSPIQPVELPVSIVSLPVYHKH
TFIVLYVNFEHRRGPGKCYNCRPAVINITLANFNETKGPLCVDTSHFTTQFVDNVKLARWSASINTGNCPFSFG
KVNNFVKFGSVCFSLKDIPGGCAMPIMANLVNSKSHNIGSLYVSWSDGDVITGVPKPVEGVSSFMNVTLNKC
TKYNIYDVSGVGVIRISNDTFLNGITYTSTSGNLLGFKDVTNGTIYSITPCNPPDQLVVYQQAVVGAMLSENFTS
YGFSNVVEMPKFFYASNGTYNCTDAVLTYSSFGVCADGSIIAVQPRNVSYDSVSAIVTANLSIPFNWTTSVQVE
YLQITSTPIVVDCSTYVCNGNVRCVELLKQYTSACKTIEDALRNSAMLESADVSEMLTFDKKAFTLANVSSFGDY
NLSSVIPSLPRSGSRVAGRSAIEDILFSKLVTSGLGTVDADYKKCTKGLSIADLACAQYYNGIMVLPGVADAERM

AMYTGSLIGGIALGGLTSAASIPFSLAIQSRLNYVALQTDVLQENQRILAASFNKAMTNIVDAFTGVNDAITQTS
QALQTVATALNKIQDVVNQQGNSLNHLTSQLRQNFQAISSSIQAIYDRLDPPQADQQVDRLITGRLAALNVFV
SHTLTKYTEVRASRQLAQQKVNECVKSQSKRYGFCGNGTHIFSLVNAAPEGLVFLHTVLLPTQYKDVEAWSGL
CVDGINGYVLRQPNLALYKEGNYYRITSRIMFEPRIPTIADFVQIENCNVTFVNISRSELQTIVPEYIDVNKTLQEL
SYKLPNYTVPDLVVEQYNQTILNLTSEISTLENKSAELNYTVQKLQTLIDNINSTLVDLKWLNRVETGSGYIPEAP
RDGQAYVRKDGEWVLLSTFLGRSLEVLFQGPGHHHHHHHHSAWSHPQFEKGGGSGGGGSGGSAWSHPQ
FEK

OC43_S-2P:

MFLILLISLPTAFAVIGDLKCPLDSRTGSLNNIDTGPPSISTATVDVTNGLGTYYVLDRVYLNTTLFLNGYYPTSGS
TYRNMALKGTDKLSTLWFKPPFLSDFINGIFAKVKNTKVFKDGVMYSEFPAITIGSTFVNTSYSVVVQPRTINST
QDGVNKLQGLLEVSVCQYNMCEYPHTICHPKLGNHFKELWHMDTGVVSCLYKRNFTYDVNATYLYFHFYQE
GGTFYAYFTDTGVVTKFLFNVYLGMALSHYYVMPLTCISRRDIGFTLEYWVTPLTSRQYLLAFNQDGIIFNAVD
CMSDFMSEIKCKTQSIAPPTGVYELNGYTVQPIADVYRRKPDLPNCNIEAWLNDKSVPSPLNWERKTFSNCN
FNMSSLMSFIQADSFTCNNIDAAKIYGMCFSSITIDKFAIPNGRKVDLQLGNLGYLQSFNYRIDTTATSCQLYYN
LPAANVSVSRFNPSTWNKRFGFIENSVFKPQPAGVLTNHDVVYAQHCFKAPKNFCPCKLNSSLCVGSGPGKN
NGIGTCPAGTNYLTCHNLCNPDPITFTGPYKCPQTKSLVGIGEHCSGLAVKSDYCGGNPCTCQPQAFLGWSAD
SCLQGDKCNIFANLILHDVNSGLTCSTDLQKANTDIKLGVCVNYDLYGISGQGIFVEVNATYYNSWQNLLYDSN
GNLYGFRDYITNRTFMIRSCYSGRVSAAFHANSSEPALLFRNIKCNYVFNNSLIRQLQPINYFDSYLGCVVNAYN
STAISVQTCDLTVGSGYCVDYSKNRRSRRAITTGYRFTNFEPFTVNSVNDSLEPVGGLYEIQIPSEFTIGNMEEFI
QTSSPKVTIDCAAFVCGDYAACKSQLVEYGSFCDNINAILTEVNELLDTTQLQVANSLMNGVTLSTKLKDGVNF
NVDDINFSSVLGCLGSECSKASSRSAIEDLLFDKVKLSDVGFVAAYNNCTGGAEIRDLICVQSYKGIKVLPPLLSE
NQISGYTLAATSASLFPPWTAAAGVPFYLNVQYRINGLGVTMDVLSQNQKLIANAFNNALDAIQEGFDATNS
ALVKIQAVVNANAEALNNLLQQLSNRFGAISSSLQEILSRLDPPEAEAQIDRLINGRLTALNAYVSQQLSDSTLVK
FSAAQAMEKVNECVKSQSSRINFCGNGNHIISLVQNAPYGLYFIHFSYVPTKYVTAKVSPGLCIAGDRGIAPKSG
YFVNVNNTWMYTGSGYYYPEPITENNVVVMSTCAVNYTKAPYVMLNTSTPNLPDFREELDQWFKNQTSVA
PDLSLDYINVTFLDLQVEMNRLQEAIKVLNGSGYIPEAPRDGQAYVRKDGEWVLLSTFLGRSLEVLFQGPGHH
HHHHHHSAWSHPQFEKGGGSGGGGSGGSAWSHPQFEK

NL63_S-2P:

MKLFLILLVLPLASCFFTCNSNANLSMLQLGVPDNSSTIVTGLLPTHWFCANQSTSVYSANGFFYIDVGNHRSA
FALHTGYYDANQYYIYVTNEIGLNASVTLKICKFSRNTTFDFLSNASSSFDCIVNLLFTEQLGAPLGITISGETVRL
HLYNVTRTFYVPAAYKLTKLSVKCYFNYSCVFSVVNATVTVNVTTHNGRVVNYTVCDDCNGYTDNIFSVQQDG
RIPNGFPFNNWFLLTNGSTLVDGVSRLYQPLRLTCLWPVPGLKSSTGFVYFNATGSDVNCNGYQHNSVVDVM
RYNLNFSANSLDNLKSGVIVFKTLQYDVLFYCSNSSSGVLDTTIPFGPSSQPYYCFINSTINTTHVSTFVGILPPTV
REIVVARTGQFYINGFKYFDLGFIEAVNFNVTTASATDFWTVAFATFVDVLVNVSATNIQNLLYCDSPFEKLQCE
HLQFGLQDGFYSANFLDDNVLPETYVALPIYYQHTDINFTATASFGGSCYVCKPHQVNISLNGNTSVCVRTSHF
SIRYIYNRVKSGSPGDSSWHIYLKSGTCPFSFSKLNNFQKFKTICFSTVEVPGSCNFPLEATWHYTSYTIVGALYV
TWSEGNSITGVPYPVSGIREFSNLVLNNCTKYNIYDYVGTGIIRSSNQSLAGGITYVSNSGNLLGFKNVSTGNIFI
VTPCNQPDQVAVYQQSIIGAMTAVNESRYGLQNLLQLPNFYYVSNGGNNCTTAVMTYSNFGICADGSLIPVR
PRNSSDNGISAIITANLSIPSNWTTSVQVEYLQITSTPIVVDCATYVCNGNPRCKNLLKQYTSACKTIEDALRLSA
HLETNDVSSMLTFDSNAFSLANVTSFGDYNLSSVLPQRNIRSSRIAGRSALEDLLFSKVVTSGLGTVDVDYKSCT
KGLSIADLACAQYYNGIMVLPGVADAERMAMYTGSLIGGMVLGGLTSAAAIPFSLALQARLNYVALQTDVLQ
ENQKILAASFNKAINNIVASFSSVNDAITQTAEAIHTVTIALNKIQDVVNQQGSALNHLTSQLRHNFQAISNSIQ
AIYDRLDPPQADQQVDRLITGRLAALNAFVSQVLNKYTEVRGSRRLAQQKINECVKSQSNRYGFCGNGTHIFS
IVNSAPDGLLFLHTVLLPTDYKNVKAWSGICVDGIYGYVLRQPNLVLYSDNGVFRVTSRIMFQPRLPVLSDFVQ

IYNCNVTFVNISRVELHTVIPDYVDVNKTLQEFAQNLPKYVKPNFDLTPFNLTYLNLSSELKQLEAKTASLFQTTV
ELQGLIDQINSTYVDLKLLNRFENGSGYIPEAPRDGQAYVRKDGEWVLLSTFLGRSLEVLFQGPGHHHHHHH
HSAWSHPQFEKGGGSGGGGSGGSAWSHPQFEK
SARS_Linker_dn5b:
MFIFLLFLTLTSGSDLDRCTTFDDVQAPNYTQHTSSMRGVYYPDEIFRSDTLYLTQDLFLPFYSNVTGFHTINHTF
GNPVIPFKDGIYFAATEKSNVVRGWVFGSTMNNKSQSVIIINNSTNVVIRACNFELCDNPFFAVSKPMGTQTH
TMIFDNAFNCTFEYISDAFSLDVSEKSGNFKHLREFVFKNKDGFLYVYKGYQPIDVVRDLPSGFNTLKPIFKLPL
GINITNFRAILTAFSPAQDIWGTSAAAYFVGYLKPTTFMLKYDENGTITDAVDCSQNPLAELKCSVKSFEIDKGIY
QTSNFRVVPSGDVVRFPNITNLCPFGEVFNATKFPSVYAWERKKISNCVADYSVLYNSTFFSTFKCYGVSATKLN
DLCFSNVYADSFVVKGDDVRQIAPGQTGVIADYNYKLPDDFMGCVLAWNTRNIDATSTGNYNYKYRYLRHG
KLRPFERDISNVPFSPDGKPCTPPALNCYWPLNDYGFYTTTGIGYQPYRVVVLSFELLNAPATVCGPKLSTDLIK
NQCVNFNFNGLTGTGVLTPSSKRFQPFQQFGRDVSDFTDSVRDPKTSEILDISPCAFGGVSVITPGTNASSEVA
VLYQDVNCTDVSTAIHADQLTPAWRIYSTGNNVFQTQAGCLIGAEHVDTSYECDIPIGAGICASYHTVSLLRSTS
QKSIVAYTMSLGADSSIAYSNNTIAIPTNFSISITTEVMPVSMAKTSVDCNMYICGDSTECANLLLQYGSFCTQL
NRALSGIAAEQDRNTREVFAQVKQMYKTPTLKYFGGFNFSQILPDPLKPTKRSFIEDLLFNKVTLADAGFMKQ
YGECLGDINARDLICAQKFNGLTVLPPLLTDDMIAAYTAALVSGTATAGWTFGAGAALQIPFAMQMAYRFNGI
GVTQNVLYENQKQIANQFNKAISQIQESLTTTSTALGKLQDVVNQNAQALNTLVKQLSSNFGAISSVLNDILSR
LDPPEAEVQIDRLITGRLQSLQTYVTQQLIRAAEIRASANLAATKMSECVLGQSKRVDFCGKGYHLMSFPQAA
PHGVVFLHVTYVPSQERNFTTAPAICHEGKAYFPREGVFVFNGTSWFITQRNFFSPQIITTDNTFVSGNCDVVI
GIINNTVYDPLQPELDSFKEELDKYFKNHTSPDVDLGDISGINASVVNIQKEIDRLNEVAKNLNESLIDLQELGKY
EQGGPGGEEAELAYLLGELAYKLGEYRIAIRAYRIALKRDPNNAEAWYNLGNAYYKQGRYREAIEYYQKALELD
PNNAEAWYNLGNAYYERGEYEEAIEYYRKALRLDPNNADAMQNLLNAKMREEGGWELQHHHHHH
HKU1_Linker_dn5b:
MFLIIFILPTTLAVIGDFNCTNSFINDYNKTIPRISEDVVDVSLGLGTYYVLNRVYLNTTLLFTGYFPKSGANFRDL
ALKGSIYLSTLWYKPPFLSDFNNGIFSKVKNTKLYVNNTLYSEFSTIVIGSVFVNTSYTIVVQPHNGILEITACQYT
MCEYPHTVCKSKGSIRNESWHIDSSEPLCLFKKNFTYNVSADWLYFHFYQERGVFYAYYADVGMPTTFLFSLYL
GTILSHYYVMPLTCNAISSNTDNETLEYWVTPLSRRQYLLNFDEHGVITNAVDCSSSFLSEIQCKTQSFAPNTGV
YDLSGFTVKPVATVYRRIPNLPDCDIDNWLNNVSVPSPLNWERRIFSNCNFNLSTLLRLVHVDSFSCNNLDKSK
IFGSCFNSITVDKFAIPNRRRDDLQLGSSGFLQSSNYKIDISSSSCQLYYSLPLVNVTINNFNPSSWNRRYGFGSF
NLSSYDVVYSDHCFSVNSDFCPCADPSVVNSCAKSKPPSAICPAGTKYRHCDLDTTLYVKNWCRCSCLPDPIST
YSPNTCPQKKVVVGIGEHCPGLGINEEKCGTQLNHSSCFCSPDAFLGWSFDSCISNNRCNIFSNFIFNGINSGT
TCSNDLLYSNTEISTGVCVNYDLYGITGQGIFKEVSAAYYNNWQNLLYDSNGNIIGFKDFLTNKTYTILPCYSGRV
SAAFYQNSSSPALLYRNLKCSYVLNNISFISQPFYFDSYLGCVLNAVNLTSYSVSSCDLRMGSGFCIDYALPSSGG
SGSGISSPYRFVTFEPFNVSFVNDSVETVGGLFEIQIPTNFTIAGHEEFIQTSSPKVTIDCSAFVCSNYAACHDLLS
EYGTFCDNINSILNEVNDLLDITQLQVANALMQGVTLSSNLNTNLHSDVDNIDFKSLLGCLGSQCGSSSRSLLE
DLLFNKVKLSDVGFVEAYNNCTGGSEIRDLLCVQSFNGIKVLPPILSETQISGYTTAATVAAMFPPWSAAAGVP
FSLNVQYRINGLGVTMDVLNKNQKLIANAFNKALLSIQNGFTATNSALAKIQSVVNANAQALNSLLQQLFNKF
GAISSSLQEILSRLDPPEAQVQIDRLINGRLTALNAYVSQQLSDITLIKAGASRAIEKVNECVKSQSPRINFCGNG
NHILSLVQNAPYGLLFIHFSYKPTSFKTVLVSPGLCLSGDRGIAPKQGYFIKQNDSWMFTGSSYYYPEPISDKNV
VFMNSCSVNFTKAPFIYLNNSIPNLSDFEAELSLWFKNHTSIAPNLTFNSHINATFLDLYYEMNVIQESIKSLNGE
EAELAYLLGELAYKLGEYRIAIRAYRIALKRDPNNAEAWYNLGNAYYKQGRYREAIEYYQKALELDPNNAEAWY
NLGNAYYERGEYEEAIEYYRKALRLDPNNADAMQNLLNAKMREEGGWELQHHHHHH
OC43_Linker_dn5b:

MFLILLISLPTAFAVIGDLKCPLDSRTGSLNNIDTGPPSISTATVDVTNGLGTYYVLDRVYLNTTLFLNGYYPTSGS
TYRNMALKGTDKLSTLWFKPPFLSDFINGIFAKVKNTKVFKDGVMYSEFPAITIGSTFVNTSYSVVVQPRTINST
QDGVNKLQGLLEVSVCQYNMCEYPHTICHPKLGNHFKELWHMDTGVVSCLYKRNFTYDVNATYLYFHFYQE
GGTFYAYFTDTGVVTKFLFNVYLGMALSHYYVMPLTCISRRDIGFTLEYWVTPLTSRQYLLAFNQDGIIFNAVD
CMSDFMSEIKCKTQSIAPPTGVYELNGYTVQPIADVYRRKPDLPNCNIEAWLNDKSVPSPLNWERKTFSNCN
FNMSSLMSFIQADSFTCNNIDAAKIYGMCFSSITIDKFAIPNGRKVDLQLGNLGYLQSFNYRIDTTATSCQLYYN
LPAANVSVSRFNPSTWNKRFGFIENSVFKPQPAGVLTNHDVVYAQHCFKAPKNFCPCKLNSSLCVGSGPGKN
NGIGTCPAGTNYLTCHNLCNPDPITFTGPYKCPQTKSLVGIGEHCSGLAVKSDYCGGNPCTCQPQAFLGWSAD
SCLQGDKCNIFANLILHDVNSGLTCSTDLQKANTDIKLGVCVNYDLYGISGQGIFVEVNATYYNSWQNLLYDSN
GNLYGFRDYITNRTFMIRSCYSGRVSAAFHANSSEPALLFRNIKCNYVFNNSLIRQLQPINYFDSYLGCVVNAYN
STAISVQTCDLTVGSGYCVDYSKNRRSRRAITTGYRFTNFEPFTVNSVNDSLEPVGGLYEIQIPSEFTIGNMEEFI
QTSSPKVTIDCAAFVCGDYAACKSQLVEYGSFCDNINAILTEVNELLDTTQLQVANSLMNGVTLSTKLKDGVNF
NVDDINFSSVLGCLGSECSKASSRSAIEDLLFDKVKLSDVGFVAAYNNCTGGAEIRDLICVQSYKGIKVLPPLLSE
NQISGYTLAATSASLFPPWTAAAGVPFYLNVQYRINGLGVTMDVLSQNQKLIANAFNNALDAIQEGFDATNS
ALVKIQAVVNANAEALNNLLQQLSNRFGAISSSLQEILSRLDPPEAEAQIDRLINGRLTALNAYVSQQLSDSTLVK
FSAAQAMEKVNECVKSQSSRINFCGNGNHIISLVQNAPYGLYFIHFSYVPTKYVTAKVSPGLCIAGDRGIAPKSG
YFVNVNNTWMYTGSGYYYPEPITENNVVVMSTCAVNYTKAPYVMLNTSTPNLPDFREELDQWFKNQTSVA
PDLSLDYINVTFLDLQVEMNRLQEAIKVLNGGEEAELAYLLGELAYKLGEYRIAIRAYRIALKRDPNNAEAWYNL
GNAYYKQGRYREAIEYYQKALELDPNNAEAWYNLGNAYYERGEYEEAIEYYRKALRLDPNNADAMQNLLNAK
MREEGGWELQHHHHHH
MERS_Linker_dn5b:
MIHSVFLLMFLLTPTESYVDVGPDSVKSACIEVDIQQTFFDKTWPRPIDVSKADGIIYPQGRTYSNITITYQGLFP
YQGDHGDMYVYSAGHATGTTPQKLFVANYSQDVKQFANGFVVRIGAAANSTGTVIISPSTSATIRKIYPAFML
GSSVGNFSDGKMGRFFNHTLVLLPDGCGTLLRAFYCILEPRSGNHCPAGNSYTSFATYHTPATDCSDGNYNRN
ASLNSFKEYFNLRNCTFMYTYNITEDEILEWFGITQTAQGVHLFSSRYVDLYGGNMFQFATLPVYDTIKYYSIIPH
SIRSIQSDRKAWAAFYVYKLQPLTFLLDFSVDGYIRRAIDCGFNDLSQLHCSYESFDVESGVYSVSSFEAKPSGSV
VEQAEGVECDFSPLLSGTPPQVYNFKRLVFTNCNYNLTKLLSLFSVNDFTCSQISPAAIASNCYSSLILDYFSYPLS
MKSDLSVSSAGPISQFNYKQSFSNPTCLILATVPHNLTTITKPLKYSYINKCSRFLSDDRTEVPQLVNANQYSPCV
SIVPSTVWEDGDYYRKQLSPLEGGGWLVASGSTVAMTEQLQMGFGITVQYGTDTNSVCPKLEFANDTKIASQ
LGNCVEYSLYGVSGRGVFQNCTAVGVRQQRFVYDAYQNLVGYYSDDGNYYCLRACVSVPVSVIYDKETKTHAT
LFGSVACEHISSTMSQYSRSTRSMLKRRDSTYGPLQTPVGCVLGLVNSSLFVEDCKLPLGQSLCALPDTPSTLTP
ASVGSVPGEMRLASIAFNHPIQVDQLNSSYFKLSIPTNFSFGVTQEYIQTTIQKVTVDCKQYVCNGFQKCEQLL
REYGQFCSKINQALHGANLRQDDSVRNLFASVKSSQSSPIIPGFGGDFNLTLLEPVSISTGSRSARSAIEDLLFDK
VTIADPGYMQGYDDCMQQGPASARDLICAQYVAGYKVLPPLMDVNMEAAYTSSLLGSIAGVGWTAGLSSFA
AIPFAQSIFYRLNGVGITQQVLSENQKLIANKFNQALGAMQTGFTTTNEAFHKVQDAVNNNAQALSKLASEL
SNTFGAISASIGDIIQRLDPPEQDAQIDRLINGRLTTLNAFVAQQLVRSESAALSAQLAKDKVNECVKAQSKRSG
FCGQGTHIVSFVVNAPNGLYFMHVGYYPSNHIEVVSAYGLCDAANPTNCIAPVNGYFIKTNNTRIVDEWSYT
GSSFYAPEPITSLNTKYVAPQVTYQNISTNLPPPLLGNSTGIDFQDELDEFFKNVSTSIPNFGSLTQINTTLLDLTY
EMLSLQQVVKALNESYIDLKELGNYTYGSGEEAELAYLLGELAYKLGEYRIAIRAYRIALKRDPNNAEAWYNLGN
AYYKQGRYREAIEYYQKALELDPNNAEAWYNLGNAYYERGEYEEAIEYYRKALRLDPNNADAMQNLLNAKM
REEGGWELQHHHHHH
SARS-2_Linker_dn5b:
MFVFLVLLPLVSSQCVNLTTRTQLPPAYTNSFTRGVYYPDKVFRSSVLHSTQDLFLPFFSNVTWFHAIHVSGTN
GTKRFDNPVLPFNDGVYFASTEKSNIIRGWIFGTTLDSKTQSLLIVNNATNVVIKVCEFQFCNDPFLGVYYHKN

NKSWMESEFRVYSSANNCTFEYVSQPFLMDLEGKQGNFKNLREFVFKNIDGYFKIYSKHTPINLVRDLPQGFS
ALEPLVDLPIGINITRFQTLLALHRSYLTPGDSSSGWTAGAAAYYVGYLQPRTFLLKYNENGTITDAVDCALDPLS
ETKCTLKSFTVEKGIYQTSNFRVQPTESIVRFPNITNLCPFGEVFNATRFASVYAWNRKRISNCVADYSVLYNSAS
FSTFKCYGVSPTKLNDLCFTNVYADSFVIRGDEVRQIAPGQTGKIADYNYKLPDDFTGCVIAWNSNNLDSKVG
GNYNYLYRLFRKSNLKPFERDISTEIYQAGSTPCNGVEGFNCYFPLQSYGFQPTNGVGYQPYRVVVLSFELLHA
PATVCGPKKSTNLVKNKCVNFNFNGLTGTGVLTESNKKFLPFQQFGRDIADTTDAVRDPQTLEILDITPCSFGG
VSVITPGTNTSNQVAVLYQDVNCTEVPVAIHADQLTPTWRVYSTGSNVFQTRAGCLIGAEHVNNSYECDIPIG
AGICASYQTQTNSPGSASSVASQSIIAYTMSLGAENSVAYSNNSIAIPTNFTISVTTEILPVSMTKTSVDCTMYIC
GDSTECSNLLLQYGSFCTQLNRALTGIAVEQDKNTQEVFAQVKQIYKTPPIKDFGGFNFSQILPDPSKPSKRSFIE
DLLFNKVTLADAGFIKQYGDCLGDIAARDLICAQKFNGLTVLPPLLTDEMIAQYTSALLAGTITSGWTFGAGAA
LQIPFAMQMAYRFNGIGVTQNVLYENQKLIANQFNSAIGKIQDSLSSTASALGKLQDVVNQNAQALNTLVKQ
LSSNFGAISSVLNDILSRLDPPEAEVQIDRLITGRLQSLQTYVTQQLIRAAEIRASANLAATKMSECVLGQSKRVD
FCGKGYHLMSFPQSAPHGVVFLHVTYVPAQEKNFTTAPAICHDGKAHFPREGVFVSNGTHWFVTQRNFYEP
QIITTDNTFVSGNCDVVIGIVNNTVYDPLQPELDSFKEELDKYFKNHTSPDVDLGDISGINASVVNIQKEIDRLN
EVAKNLNESLIDLQELGKYEQGSGEEAELAYLLGELAYKLGEYRIAIRAYRIALKRDPNNAEAWYNLGNAYYKQG
RYREAIEYYQKALELDPNNAEAWYNLGNAYYERGEYEEAIEYYRKALRLDPNNADAMQNLLNAKMREEGGW
ELQHHHHHH

Supplementary Fig. 8: Construct list and amino acid sequences for CoV_S-2P and _dn5 constructs.

Recombinant spike protein sequences derived from the indicated coronavirus prefix. S-2P suffix indicates that the construct is a soluble prefusion-stabilized spike protein using two-proline mutations. The _dn5b suffix indicates that the prefusion-stabilized spikes are displayed on the I53_dn5 scaffold through genetic fusion to the I53_dn5b component. Linker indicates a short flexible linker between the spike and I53_dn5b scaffold component.
