# Supplementary material for: Nanoparticle display of prefusion coronavirus spike elicits S1-focused cross-reactive antibody response against diverse coronavirus subgenera
Source: Nat Commun. 2023 Oct 4;14:6195. doi: 10.1038/s41467-023-41661-4 (PMC10551005; doi:10.1038/s41467-023-41661-4)
Supplement: Supplementary file 3 — Reporting Summary [file 41467_2023_41661_MOESM3_ESM.pdf]

nature portfolio

Corresponding author(s): Kizzmekia S Corbett

Last updated by author(s): Jul 31, 2023

Reporting Summary

Nature Portfolio wishes to improve the reproducibility of the work that we publish. This form provides structure for consistency and transparency in reporting. For further information on Nature Portfolio policies, see our Editorial Policies and the Editorial Policy Checklist.

Statistics

For all statistical analyses, confirm that the following items are present in the figure legend, table legend, main text, or Methods section.

n/a	Confirmed	
☐	☒	The exact sample size (*n*) for each experimental group/condition, given as a discrete number and unit of measurement
☐	☒	A statement on whether measurements were taken from distinct samples or whether the same sample was measured repeatedly
☐	☒	The statistical test(s) used AND whether they are one- or two-sided<br>*Only common tests should be described solely by name; describe more complex techniques in the Methods section.*
☒	☐	A description of all covariates tested
☐	☒	A description of any assumptions or corrections, such as tests of normality and adjustment for multiple comparisons
☐	☒	A full description of the statistical parameters including central tendency (e.g. means) or other basic estimates (e.g. regression coefficient) AND variation (e.g. standard deviation) or associated estimates of uncertainty (e.g. confidence intervals)
☐	☒	For null hypothesis testing, the test statistic (e.g. *F*, *t*, *r*) with confidence intervals, effect sizes, degrees of freedom and *P* value noted<br>*Give P values as exact values whenever suitable.*
☒	☐	For Bayesian analysis, information on the choice of priors and Markov chain Monte Carlo settings
☒	☐	For hierarchical and complex designs, identification of the appropriate level for tests and full reporting of outcomes
☒	☐	Estimates of effect sizes (e.g. Cohen's *d*, Pearson's *r*), indicating how they were calculated

Our web collection on statistics for biologists contains articles on many of the points above.

Software and code

Policy information about availability of computer code

Data collection	Negative Stain Electron Microscopy data was collected using EPU software version 2.12.1.2782REL (ThermoFisher). Images of particles were picked automatically using EMAN2 or in-house written software (Yaroslav Tsybovsky, unpublished).
Data analysis	RELION 3.1 was used for 2D and 3D refinement of NSEM datasets<br>Prism v9 was used for serological data analysis and statistics.<br>ChimeraX 1.3 and Pymol v2.5 were used for analysis of 3D reconstruction of NSEM.

For manuscripts utilizing custom algorithms or software that are central to the research but not yet described in published literature, software must be made available to editors and reviewers. We strongly encourage code deposition in a community repository (e.g. GitHub). See the Nature Portfolio guidelines for submitting code & software for further information.

Data

Policy information about availability of data

All manuscripts must include a data availability statement. This statement should provide the following information, where applicable:

- Accession codes, unique identifiers, or web links for publicly available datasets
- A description of any restrictions on data availability
- For clinical datasets or third party data, please ensure that the statement adheres to our policy

The data supporting the findings of this study are available within the paper and its supplementary files. Source data will be made available in a publicly accessible

The data supporting the findings of this study are available within the paper and its supplementary files. Source data will be made available in a publicly accessible repository. Structures for 3D map overlays: PDB: 5X5F [https://doi.org/10.2210/pdb5x5f/pdb], PDB: 6PXH [https://doi.org/10.2210/pdb6pxh/pdb]

Human research participants

Policy information about studies involving human research participants and Sex and Gender in Research.

Reporting on sex and gender	No human research participants were used in this study
Population characteristics	No human research participants were used in this study
Recruitment	No human research participants were used in this study
Ethics oversight	No human research participants were used in this study

Note that full information on the approval of the study protocol must also be provided in the manuscript.

Field-specific reporting

Please select the one below that is the best fit for your research. If you are not sure, read the appropriate sections before making your selection.

☒ Life sciences ☐ Behavioural & social sciences ☐ Ecological, evolutionary & environmental sciences

For a reference copy of the document with all sections, see nature.com/documents/nr-reporting-summary-flat.pdf

Life sciences study design

All studies must disclose on these points even when the disclosure is negative.

Sample size	Sample sizes were determined based on experience with similar experimental designs and publications. A group size of 10 animals will give 89% power to detect 2-fold differences of 49% power to detect 1.5-fold differences between vaccine groups (set for 4) in the magnitude of the immunological parameters based on a two-tailed test of means with alpha set to 0.05 (calculation was performed by 1-way ANOVA tools at powerandsamplesize.com).
Data exclusions	No data were excluded.
Replication	Animal studies were performed at least twice with N=5-10 per group with equal sample sizes for per group. All immunoassays (antibody binding, neutralization, and depletion experiments) were performed in technical duplicate or triplicate with at least 1 repeat. Reported differences remained consistent across each repeat. Great efforts were made to repeat the MERS challenge and EMPEM studies, unfortunately pandemic-related shipping, transportation, and operational capacity challenges prevented us from doing so. However, these experiments will be repeated for future projects related to pan-coronavirus vaccine development.
Randomization	All mice were allocated randomly. all other experiments were for characterization purposes and not randomized.
Blinding	Blinding was not done as assays were performed by the same team that immunized animals.

Reporting for specific materials, systems and methods

We require information from authors about some types of materials, experimental systems and methods used in many studies. Here, indicate whether each material, system or method listed is relevant to your study. If you are not sure if a list item applies to your research, read the appropriate section before selecting a response.

Materials & experimental systems

n/a	Involved in the study	
☐	☒	Antibodies
☐	☒	Eukaryotic cell lines
☒	☐	Palaeontology and archaeology
☐	☒	Animals and other organisms
☒	☐	Clinical data
☒	☐	Dual use research of concern

Methods

n/a	Involved in the study	
☒	☐	ChIP-seq
☒	☐	Flow cytometry
☒	☐	MRI-based neuroimaging

Antibodies

Antibodies used	JC57-13 - mAb specific for MERS Spike<br>CR6261 - mAb specific for group 1 influenza hemagglutinin<br>S652-H9 - mAb specific for SARS spike<br>Ly-Cov-555 - mAb specific for SARS-CoV-2 spike<br>Horseradish Peroxidase (HRP)-conjugate anti-mouse IgG (Southern Biotech, Birmingham, AL) was used for all mouse ELISAs.<br>HRP-conjugated anti-human IgG (Southern Biotech) was used for antigenicity ELISAs using human mAbs as applicable
Validation	Antibodies generated in-house were validated by ELISA with their specific antigen to assess binding activity prior to use in experiments.

Eukaryotic cell lines

Policy information about cell lines and Sex and Gender in Research

Cell line source(s)	Expi293F (ThermoFisher, Cat#: A14527), Freestyle 293F (ThermoFisher, Cat#: R79007), Adherent HEK293T/17 (ATCC CRL-11268). 293T-ACE2 stable cell line: ACE2-expressing 293T cells (provided by M. Farzan, Scripps Research Institute).
Authentication	Authentication for all cell lines performed by manufacturers. No further authentication was performed by authors.
Mycoplasma contamination	All cell lines tested negative for mycoplasma.
Commonly misidentified lines (See ICLAC register)	No commonly misidentified lines used.

Animals and other research organisms

Policy information about studies involving animals; ARRIVE guidelines recommended for reporting animal research, and Sex and Gender in Research

Laboratory animals	Female BALB/cJ and C57BL/6J mice from The Jackson Laboratory were used for all animal studies except for MERS challenge which used male and female 288/330+/+ mice (derived from C57BL/6 background). UNC mice: 12-hour light/dark cycle (7am-7pm), temperature between 20 and 23C, humidity 30-70% (usually in the 50s).<br><br>VRC mice: 12-hour light/dark cycle (6am-6pm), temperature between 22 and 25C, humidity 30-70% (usually around 35%)<br><br>For immunogenicity studies, female BALB/cJ or C57BL/6 mice aged 6- to 8-weeks (Jackson Laboratory) were used. For challenge studies, 16- to 20-week-old male and female 288/330+/+ mice21 were used.
Wild animals	This study did not use wild animals.
Reporting on sex	Female mice were used for all experiments except for MERS challenge which used both male and female mice.
Field-collected samples	The study did not use field-collected samples.
Ethics oversight	Animal experiments were carried out in compliance with US National Institutes of Health regulations and approval from the Animal Care and Use Committee of the Vaccine Research Center and the Institutional Animal Care and Use Committee of the University of North Carolina at Chapel Hill.

Note that full information on the approval of the study protocol must also be provided in the manuscript.
